# Phosphatidylserine treatment relieves the block to retrovirus infection of cells expressing glycosylated virus receptors

**DOI:** 10.1186/1742-4690-2-49

**Published:** 2005-08-09

**Authors:** David A Coil, A Dusty Miller

**Affiliations:** 1 Division of Human Biology, Fred Hutchinson Cancer Research Center, Seattle, Washington 98109-1024 USA; 2 Molecular and Cellular Biology Program, Fred Hutchinson Cancer Research Center, Seattle, Washington 98109-1024 USA

## Abstract

**Background:**

A major determinant of retrovirus host range is the presence or absence of appropriate cell-surface receptors required for virus entry. Often orthologs of functional receptors are present in a wide range of species, but amino acid differences can render these receptors non-functional. In some cases amino acid differences result in additional N-linked glycosylation that blocks virus infection. The latter block to retrovirus infection can be overcome by treatment of cells with compounds such as tunicamycin, which prevent the addition of N-linked oligosaccharides.

**Results:**

We have discovered that treatment of cells with liposomes composed of phosphatidylserine (PS) can also overcome the block to infection mediated by N-linked glycosylation. Importantly, this effect occurs without apparent change in the glycosylation state of the receptors for these viruses. This effect occurs with delayed kinetics compared to previous results showing enhancement of virus infection by PS treatment of cells expressing functional virus receptors.

**Conclusion:**

We have demonstrated that PS treatment can relieve the block to retrovirus infection of cells expressing retroviral receptors that have been rendered non-functional by glycosylation. These findings have important implications for the current model describing inhibition of virus entry by receptor glycosylation.

## Background

Many of the cellular receptors for retroviruses have been well characterized (for review see [1]). These receptors perform a wide variety of cellular functions and can be single-transmembrane, GPI-anchored, or multiple-membrane-spanning proteins. The presence or absence of functional receptors on the cell surface is a major determinant of virus tropism. In some cases, otherwise functional receptors are glycosylated and therefore unusable by particular retroviruses [2-6]. Since these sites of glycosylation are often near the binding sites used by viruses, glycosylation is thought to be an important defense mechanism evolved by cells in their battle against virus infection (for example see [7]).

One particularly well-studied example of glycosylation-blocked receptors involves those for the cat endogenous retrovirus RD114, which is unable to enter NIH 3T3 mouse cells unless these cells have been treated with agents, including tunicamycin, that prevent the addition of N-linked oligosaccharides to proteins in the endoplasmic reticulum. The receptor for RD114 in tunicamycin-treated NIH 3T3 cells is a multiple-membrane spanning protein called ASCT1 (standard name SLC1A4), which is a neutral amino acid transporter [5]. RD114 also uses a closely related human protein, ASCT2 (standard name SLC1A5, also called RDR) as a receptor [8,9]. Sequence differences in the mouse ortholog of human ASCT2 prevent it from serving as a receptor, even after tunicamycin treatment [5].

Other examples of glycosylation-blocked receptors are the hamster and rat orthologs of the receptor for Moloney murine leukemia virus (MoMLV), CAT1 (standard name SLC7A1). Prevention of receptor glycosylation by treatment of rat or hamster cells with tunicamycin relieves the block to infection by MoMLV [3,10]. Like ASCT1 and ASCT2, CAT1 is an amino acid transporter, in this case for lysine, arginine, and ornithine [11-13]. If the N-linked glycosylation sites of mouse ASCT1, hamster CAT1, or rat CAT1 are removed through mutagenesis, these proteins are fully functional as virus receptors [3,10,14]. To date, removal of N-linked glycosylation through either mutagenesis of the oligosaccharide attachment sites or by treatment with inhibitors of glycosylation are the only ways known to relive the block to infection by RD114 and MoMLV viruses in the respective rodent cell lines.

We recently have shown that treatment of target cells with phosphatidylserine (PS) enhances enveloped virus infection by up to 20-fold [15]. This effect is not observed with other phospholipids, and is thought to occur through an enhancement of virus fusion [15]. Importantly, in all cases tested where a functional receptor was present, PS treatment enhanced virus infection. Conversely, when a functional receptor was not present, PS treatment did not allow infection of target cells. Here we show that phosphatidylserine treatment can relieve the block to infection mediated by glycosylation-blocked receptors and further investigate this phenomenon.

## Results

### PS treatment allows infection of cell types expressing glycosylation-blocked receptors

Our previous work demonstrated that PS-dependent enhancement of infection requires functional receptors [15], and we will refer to this effect as "non-specific enhancement" of virus infection by PS. We wanted to extend these observations by examining the effects of PS on virus entry in the case where the receptor was present but was inactive due to receptor glycosylation. We used the LAPSN retroviral vector [16] that encodes human placental alkaline phosphatase (AP) as a marker for infection. Viruses carrying this vector contained Gag-Pol proteins from MoMLV and Env proteins from either MoMLV or RD114. For simplicity we will call these viruses MoMLV or RD114 vectors, respectively. MoMLV vectors are unable to enter CHO cells and RD114 vectors are unable to enter NIH 3T3 cells unless these cells are first treated with tunicamycin to prevent receptor glycosylation [3-5]. Table 1 shows that pretreatment of CHO and NIH 3T3 cells with 400 μM PS for 24 h allowed efficient entry of MoMLV and RD114 vectors, respectively. Hereafter we will refer to this effect as "glycosylation-specific enhancement" by PS, in contrast to the "non-specific enhancement" described in our previous work.

**Table 1:** PS treatment allows infection of cells expressing glycosylation-blocked retrovirus receptors^a^

Target cells	Vector	PS treatment	Vector titer (AP^+ ^FFU/ml)
NIH 3T3	RD114	-	<1
	RD114	+	2.3 × 10^4^
CHO-K1	MoMLV	-	<5
	MoMLV	+	2.3 × 10^3^

^a ^Virus infections and PS preparation were performed as described in Materials and Methods. Where indicated, cells were treated with 400 μM PS for 24 h. Data shown are the averages of three independent experiments, each done in duplicate. Values from different experiments varied no more than three-fold.

### PS treatment does not affect receptor glycosylation

**Figure 1:**
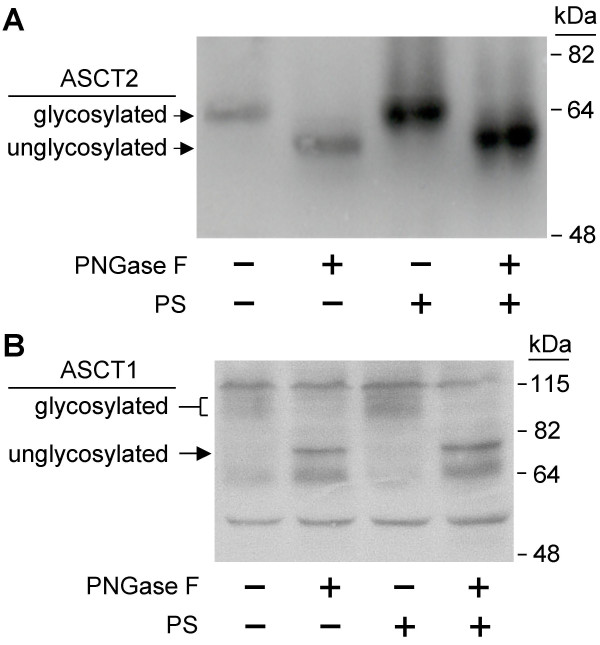
**Analysis of N-linked oligosaccharide modification of ASCT1 and ASCT2 with or without PS treatment. **(A) NIH 3T3/ASCT2 cells that express HA-tagged human ASCT2 were treated with 400 μM PS for 24 h. Cell lysates were treated with or without PNGase F as described in Materials in Methods, and lysates were analyzed by Western immunoblotting with anti HA-tag monoclonal antibody. (B) 293T cells were transiently transfected with a myc-tagged murine ASCT1 expression plasmid. 400 μM PS was added 24 h post-transfection. Cell lysates were made 48 h post-transfection, were treated with or without PNGase F as described in Materials in Methods, and were analyzed by Western immunoblotting with anti Myc-tag monoclonal antibody.

A simple explanation for these results might be that PS inhibits receptor glycosylation, as does tunicamycin treatment. As described above, murine ASCT1 functions as a receptor for RD114 in NIH 3T3 cells treated with tunicamycin [5]. Treatment of cell lysates with peptide N-glycosidase F (PNGase F) causes an increase in the electrophoretic mobility of ASCT1 as a result of removal of the N-linked glycosylation [14]. We attempted to examine the glycosylation status of a myc-tagged ASCT1 protein in NIH 3T3 cells but were unable to clearly visualize the protein due to technical problems including high background antibody binding. However, we were able to examine the glycosylation state of a hemagglutinin (HA)-tagged human ASCT2 protein in NIH 3T3/ASCT2 cells (Figure 1A). In the non-PS treated cells there was a clear increase in mobility of ASCT2 when incubated with PNGase F, demonstrating that this protein is normally glycosylated. Furthermore, none of the protein is found in the unglycosylated state prior to PNGase F treatment. The same mobility shifts were observed in cells treated with PS, indicating that treatment with PS does not affect the glycosylation state of this protein in NIH 3T3 cells.

To examine ASCT1 glycosylation directly, we transiently expressed a myc-tagged mouse ASCT1 in 293T cells and examined the effects of PS treatment on glycosylation (Figure 1B). These cells were treated with either 35 μM PS or were left untreated. This concentration of PS was chosen because it induced the highest vector infection rate in 293T cells and a high concentration of PS (400 μM) was toxic to 293T cells (data not shown). As for the HA-tagged ASCT2 protein, there was no detectable unglycosylated receptor present in the PS treated cells, indicating that ASCT1 glycosylation is unaffected by PS treatment.

### The non-specific enhancement of infection by PS treatment occurs rapidly

We have previously postulated that the non-specific enhancement of virus infection by PS occurs through an effect on virus fusion [15]. If this were true, the effect should happen relatively quickly since all that is required is for the PS liposomes to fuse with the plasma membrane of the cell and change the physical characteristics of the membrane. We undertook infections using RD114 vector on normally infectable NIH 3T3/ASCT2 cells given only a short exposure to PS, in contrast to the 24 h treatment used in previous experiments. Cells were treated with PS for 1 h, virus was added for 2 h, and the cells were trypsinized and replated. With only 1 h of PS treatment, virus infection was increased almost 4-fold. This experiment was repeated twice with the same results. While not as much as the full 10 to 20-fold increase in infection when treated for 24 h, this demonstrates that the effect of PS on virus infection is indeed rapid. However when the parental NIH 3T3 cells, containing the glycosylation-blocked receptor, were treated in the same manner, no infection by the RD114 vector was observed (data not shown).

### The non-specific and glycosylation-specific enhancements of infection have different time courses

**Figure 2:**
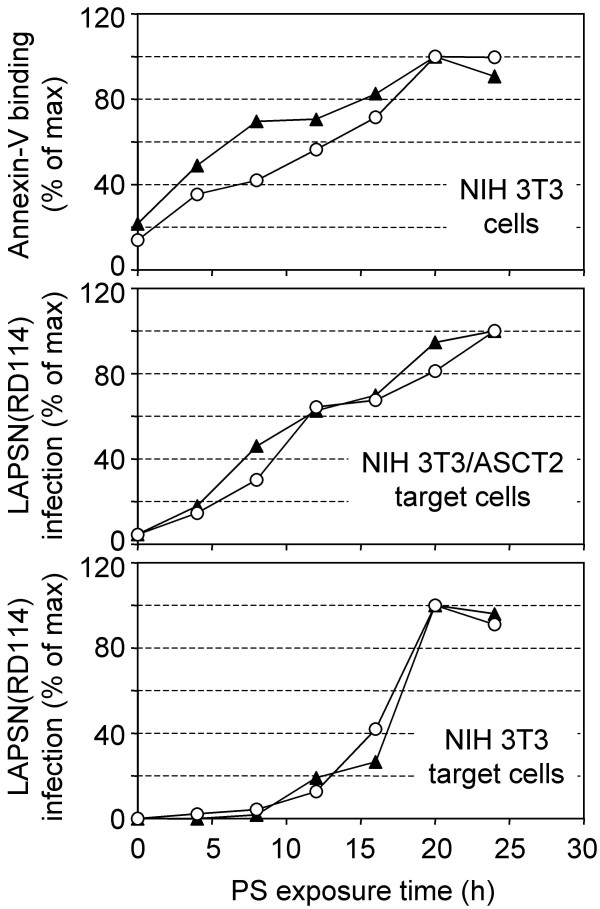
**Time course of cell-surface PS levels and cell susceptibility to RD114 vector infection of NIH 3T3/ASCT2 and NIH 3T3 cells during treatment with PS. **Cells were plated on day 0. 400 μM PS was added on day 1 at 24, 20, 16, 12, 8, and 4 h pre-infection. At the time of infection, cells were either infected with the RD114 vector [LAPSN(RD114)] or were assayed for cell-surface PS levels by using annexin-V. Top panel: annexin-V staining of NIH 3T3 cells was undertaken as described in Materials and Methods. Middle panel: LAPSN(RD114) infection of NIH 3T3/ASCT2 cells. Bottom Panel: LAPSN(RD114) infection of NIH 3T3 cells. Data points shown are means of duplicates, and each series represents an independent experiment. Data is represented as a percentage of the highest value observed.

The preceding results suggest that the glycosylation-specific enhancement of PS treatment is delayed when compared to the non-specific enhancement of virus infection. To compare these two effects we examined RD114 vector infection of both NIH 3T3 cells and NIH 3T3/ASCT2 cells over a longer time course. Cells were treated with PS at time points from 4–24 h and were then infected with the RD114 vector. The cell surface PS levels were also measured at each timepoint by annexin-V staining. We found a linear relationship between the time after PS addition and the amount of PS present in the outer leaflet of the membrane (Figure 2, top panel). Furthermore, there was a direct relationship between the amount of PS present in the membrane and infection of normally-infectable NIH 3T3/ASCT2 cells by the RD114 vector (Figure 2, middle panel). In contrast, there was a long delay in the increase in RD114 vector infection of NIH 3T3 cells following PS addition, with the major enhancement of virus infection occurring after 12 h Figure 2, bottom panel).

### Effects of PS at reduced concentrations on RD114 vector infection of NIH 3T3 cells

**Figure 3:**
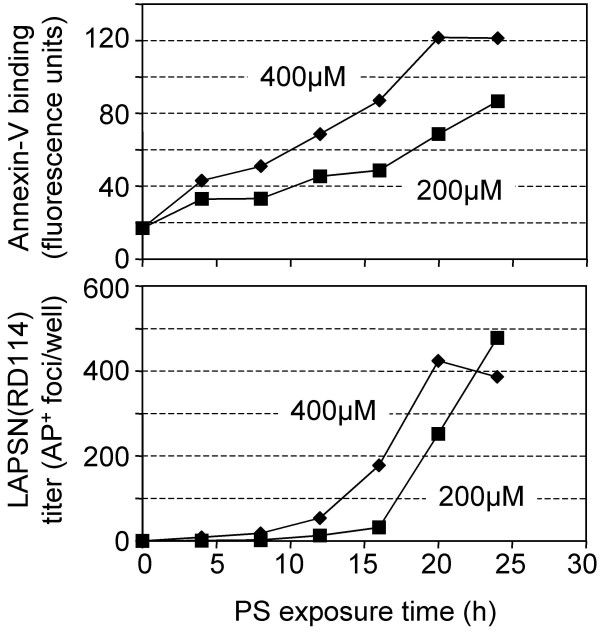
**Effects of PS at a reduced concentration on RD114 vector infection of NIH 3T3 cells. **PS liposomes were generated and added to NIH 3T3 cells at either 400 μM or 200 μM concentration. Cells were analyzed for cell-surface PS levels by using annexin-V or were infected with the RD114 vector [LAPSN(RD114)] as described in Materials and Methods. Top panel: Annexin-V staining of NIH 3T3 cells. Bottom panel: LAPSN(RD114) infection of NIH 3T3 cells. Data shown are the average of duplicates. The entire experiment was repeated with very similar results.

The long delay between addition of PS and the glycosylation-specific enhancement of virus infection suggests that a threshold amount of PS in the cell membrane may be required for the observed enhancement. To address this possibility we undertook a 24-h time course as described above, using half the amount of PS (200 μM) (Figure 3). The total amount of PS incorporated into the plasma membrane was reduced at each timepoint, and saturation did not appear to be reached. The reduced incorporation of PS had the result of increasing the delay of RD114 vector infection of NIH 3T3 cells from 12 to more than 16 h, supporting the hypothesis that a threshold amount of PS is required for the glycosylation-specific enhancement of virus infection.

### The dose-response of non-specific and glycosylation-specific enhancement of virus infection by PS differs

**Figure 4:**
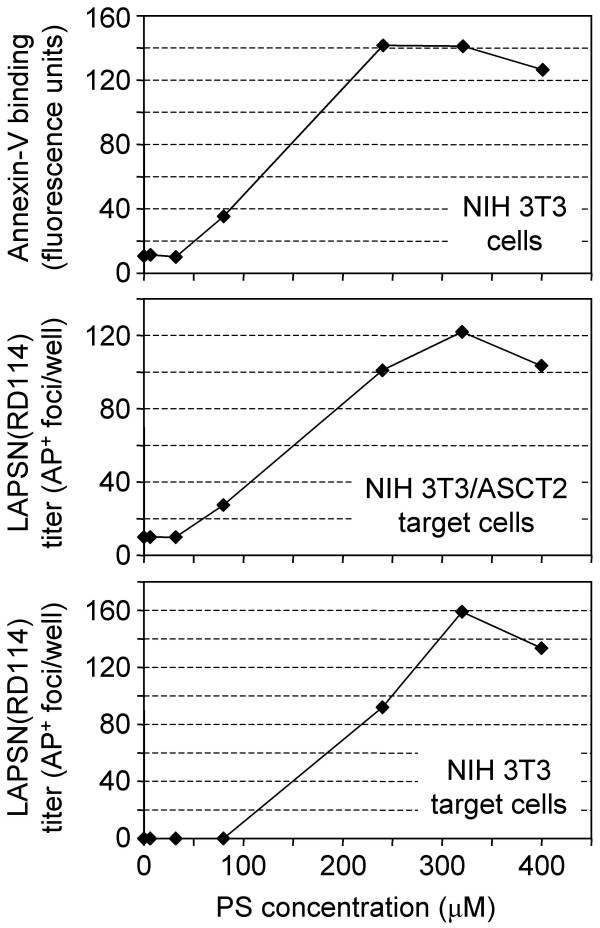
**Effects of PS concentration on cell-surface PS levels and RD114 vector infection of NIH 3T3 or NIH 3T3/ASCT2 cells. **PS liposomes were generated and added to cells at concentrations of 0, 6.4, 32, 80, 240, 320, and 400 μM. Annexin-V staining and infections were undertaken as described in Materials and Methods. Top panel: Annexin-V staining of NIH 3T3 cells. Middle panel: RD114 vector [LAPSN(RD114)] infection of NIH 3T3/ASCT2 cells. Bottom panel: LAPSN(RD114) infection of NIH 3T3 cells. Data shown are the average of duplicates. The entire experiment was repeated twice with very similar results.

It appears from the results shown in Figure 3 that there is a simple relationship between amount of PS present in the membrane and the non-specific enhancement of virus infection. We next examined the effect of 24 h treatment with various concentrations of PS on RD114 vector infection of both NIH 3T3/ASCT2 cells and NIH 3T3 cells (Figure 4). Infection and annexin-V measurements were undertaken as previously described. At very low levels of PS, which are not detectable by annexin-V, no infection on either cell type was observed. As soon as an increase in PS levels was observed, there was a corresponding increase in RD114 infection of the NIH 3T3/ASCT2 cells. However, infection of NIH 3T3 cells was not detectable until a higher concentration of PS was reached, further supporting the hypothesis of a required threshold concentration for infection through the glycosylation-specific pathway.

## Discussion

Here we report that PS treatment of target cells containing glycosylation-blocked viral receptors allows virus infection. Importantly, this occurs without removal of the oligosaccharide itself, unlike the case with tunicamycin treatment. Furthermore, this glycosylation-specific effect takes place in NIH 3T3 cells on a different timescale than the non-specific enhancement of virus infection by PS, and appears to require a threshold concentration of cell-surface PS. When NIH 3T3 cells are treated with 200 μM PS, they reach the same level of infectivity after 24 h as when treated with 400 μM PS, but take longer before infection is observable, suggesting that the observed enhancement of infection is not merely a signaling cascade initiated by the addition of PS to the cell. One explanation for such a long delay is that *de novo *protein synthesis is required for the glycosylation-specific effect of PS treatment. Additional experiments will be needed to address this question. Unfortunately, preliminary experiments have demonstrated that PS treatment combined with inhibition of protein synthesis by cycloheximide is lethal to cells (data not shown), further complicating this analysis.

Additionally we have shown that the non-specific enhancement by PS occurs rapidly, and there is a direct correlation between amount of cell-surface PS and the amount of non-specific enhancement of virus infection. This result supports our previous hypothesis that the non-specific enhancement occurs through an influence of virus fusion.

Our results suggest that the block to infection of glycosylated receptors may occur at a different stage of virus entry than previously assumed. It has been proposed that glycosylation prevents MoMLV or RD114 from binding to their cognate receptors, thereby terminating virus entry at a very early step [7]. However, our results demonstrate that these two viruses can still infect cells containing fully glycosylated receptors. However, we have not ruled out the possibility that PS might induce subtle changes in receptor glycosylation, such as alterations in the structure or branching of the N-linked oligosaccharides, that might affect virus entry.

Instead of a block to virus binding, it is possible that PS affects the packing or mobility of the receptors in the plasma membrane. Several groups have suggested that receptor clusters, or multivalent Env-receptor complexes are required for retrovirus infection [17-21]. For example, an ASLV-A virion appears to require multiple contacts with receptors in order to enter a fusogenic state [21]. It is possible that glycosylated receptors are normally unable to pack as tightly, or move through the membrane as rapidly as their unglycosylated forms in order to facilitate virus infection. In this model, the disruption to the plasma membrane caused by PS treatment could allow sufficient concentrations of receptor to contact the viral Env proteins and initiate fusion. Exogenous PS has been shown to affect the curvature and stability of a lipid bilayer, providing a mechanism for this disruption [22,23]. On the other hand, fewer receptor contacts could be required by the virus to form a fusion pore if the activation energy for fusion to occur has been lowered by PS treatment [15]. Similarly, it is possible that the glycosylation of the receptors prevents the membranes from coming in close enough contact to fuse, but that the destabilization of the plasma membrane by PS increases the distance at which this fusion can occur. Further study will be required to understand the mechanism of glycosylation-specific enhancement of virus entry through PS treatment.

## Conclusion

In summary, these results expand on our previous findings regarding the mechanism of enhancement of virus infection by PS treatment, and demonstrate an effect of PS treatment on cells containing glycosylation-blocked receptors. The ability to promote CHO-K1 and NIH 3T3 infection by MoMLV and RD114 vectors without tunicamycin treatment should be of interest to researchers studying these viruses and to those studying the nature of the glycosylation-induced block to retrovirus infection.

## Methods

### Cell culture and plasmids

NIH 3T3 thymidine kinase-deficient mouse embryo fibroblasts [24], and 293T human embryonic kidney cells [25] were maintained at 37°C and 5% CO_2 _in Dulbecco's modified Eagle medium with a high concentration of glucose (4.5 g per liter) and 10% FBS. CHO-K1 hamster cells (ATCC CCL-61) were maintained in Minimal Essential Medium Alpha at 37°C and 5% CO_2_. Clonal NIH 3T3 cells expressing an HA-tagged human ASCT2 (NIH 3T3/ASCT2 cells) were generated by transduction with the retroviral vector LNCRDRHA, that contains a human RDR (ASCT2) cDNA with a carboxy-terminal HA tag cloned into the LNCX retroviral vector [26]. The expression plasmid containing the *myc*-tagged murine ASCT1 was kindly provided by David Kabat [14].

### Virus production

LAPSN is a Moloney murine leukemia virus (MoMLV)-based vector that encodes human placental alkaline phosphatase (AP) and neomycin phosphotransferase [16]. LAPSN containing viruses were generated from the following packaging lines expressing the indicated Env proteins; FlyRD (RD114) [27], and PE501 (MoMLV) [26]. All retroviral vectors used in these studies were harvested in medium exposed to producer cells and were centrifuged at 1,000 × g for 5 min to remove cells and debris.

### Virus assays

All retrovirus vector infections were undertaken as follows. On day 0, cells were plated at 5 × 10^4 ^cells/well in 6-well dishes. On day 1, fresh phospholipid liposomes were generated and added to cells at 400 μM (unless otherwise noted). On day 2, the medium was replaced with fresh medium containing 4 μg/ml Polybrene and virus was added to the wells. On day 5 the cells were fixed with 0.5% glutaraldehyde and stained for AP expression. For the 24-h infection time courses, a large batch of PS liposomes was produced on day 1, and was added to cells every 4 h from 0–24 h. At 24 h, cells were either infected as described above or were prepared for annexin-V labeling.

### Annexin-V labeling

Alexa Fluor 488-conjugated annexin-V, propidium iodide (PI), and annexin binding buffer were obtained from the Vybrant Apoptosis Assay Kit #2 (Molecular Probes, Eugene, OR). Annexin-V labeling was performed using a slight variation of the manufacturer's protocol as previously described [28]. The geometric mean fluorescence of 10,000 cells was obtained for the unlabeled and labeled cell populations, and the mean of the unlabeled cells was subtracted from the mean of the labeled cells to determine the relative amount of cell-surface PS for each sample. Dead cells were excluded from analysis on the basis of PI staining.

### Generation of liposomes

L-α-phosphatidyl-L-serine was obtained as a 10 mg/ml solution in chloroform:methanol (95:5) (Sigma, St Louis, MO). To generate liposomes, phospholipid was dried in a glass tube under nitrogen, and resuspended in PBS to a final concentration of 5 mM. This solution was sonicated on ice 3 times for 5 min each, using a W-385 sonicator with a microtip on output level 3 (Heat Systems Ultrasonics). The liposomes were filtered through a 0.2 μm pore-size syringe filter and were used immediately unless otherwise described.

### Western blot analysis

For analysis of the HA-tagged human ASCT2, washed cells were lysed for 30 min at 4°C in lysis buffer (50 mM Tris-HCL [pH 8.0], 150 mM NaCl, and 1% NP-40), and centrifuged at 970 × g for 10 min to remove nuclei and cell debris. The supernatant was boiled for 10 min after addition of SDS and β-mercaptoethanol to final concentrations of 0.5% and 1%, respectively. The sample was divided, an equal amount of either PNGase F (New England Biolabs) or lysis buffer was added to each half, and the samples were kept at 37°C for 3 h. The treated and untreated samples were analyzed by electrophoresis in a 10% polyacrylamide gel containing 0.1% SDS. The proteins were transferred to nitrocellulose membranes, blocked in 5% powdered milk, incubated with appropriate concentrations of anti-HA primary and secondary antibodies, and visualized using a chemiluminescence kit (Amersham Biosciences). Analysis of ASCT1 was performed following transient transfection of 293T cells with a myc-tagged expression vector for murine ASCT1 [14] using the calcium phosphate method [29]. Cell lysates were collected at 48 h post-transfection and were treated as described above, followed by incubation of Western blots with appropriate concentrations of anti-Myc tag primary and secondary antibodies.

## Competing interests

The authors declare that they have no competing interests.

## Authors' contributions

DAC helped design the study, carried out the experiments, analyzed the data, and drafted the manuscript. ADM helped design the study and write the manuscript.
